# Aberrant splicing in human cancer shows possible functional impact on transcription factors

**DOI:** 10.1038/s41388-026-03876-9

**Published:** 2026-07-03

**Authors:** Khalique Newaz, Olga Tsoy, Jan Baumbach

**Affiliations:** 1 Institute for Computational Systems Biomedicine, University of Hamburg, Hamburg, Germany; 2 Department of Computer Science, Bioinformatics, Vrije Universiteit Amsterdam, Amsterdam, The Netherlands; 3 Department of Mathematics and Computer Science, University of Southern Denmark, Odense, Denmark

**Keywords:** Cancer, Transcriptomics

## Abstract

Cancer shows aberrant alternative splicing (AS), which could functionally perturb proteins required for normal cellular behavior. Transcription factors (TFs) are proteins that regulate gene transcription. Some evidence about AS-driven perturbation of TFs in cancer has been documented. A systematic analysis of how cancer-specific AS could affect structural or functional characteristics of TFs is missing. Such an analysis could reveal the molecular mechanisms of cancer progression due to transcription misregulations, and thus could help identify therapeutic targets. Here, we systematically analyzed AS-induced structural and potential functional perturbations of TFs across 15 cancer types. We identified 2170 perturbed AS events (i.e., events showing significant AS pattern differences between normal and paired cancer samples) that affect 727 TFs across 14 cancer types. In 205 TFs, the perturbed AS events affect known functional domains of TFs. Additional evidence for a potential functional impact of cancer-specific AS on TFs was also found by (1) relating AS patterns of the perturbed AS events with the DNA-binding and regulatory activity of TFs and (2) using cancer dependency data to explore whether the affected TFs are essential for cancer cell line proliferation. Our findings show a large-scale, likely functional, perturbation of TFs due to cancer-specific AS.

## Introduction

Alternative splicing (AS) results in multiple different messenger ribonucleic acids (mRNAs) originating from a single gene [1]. AS is associated with normal cellular processes, including tissue development and identity [2]. Normal AS patterns are perturbed in diseases [3], e.g., cancer [4]. Large-scale RNA sequencing (RNAseq)-based evidence for aberrant AS in cancer has been collected. For example, The Cancer Genome Atlas (TCGA) SpliceSeq database contains RNAseq-based quantification of AS events across 33 cancer types [5]. TCGASpliceSeq has been used to explore cancer-specific AS [6]. For example, Yongsheg et al. [7] investigated relationships between somatic mutations in cancer and cancer-specific AS patterns, and found that different mutations in the same gene showed distinct AS perturbations. Junyi et al. [8] hypothesized that RNA-binding proteins regulate cancer-specific AS patterns and identified such proteins as potential therapeutic targets. Similarly, Cheng et al. [9] investigated whether abnormal AS of splicing factors could explain cancer-specific AS patterns.

Aberrant AS in cancer has also been shown to impact an important group of proteins called transcription factors (TFs) [10]. TFs bind DNA to regulate transcription of genes [11]. Cancer-specific dysregulation of TFs perturbs normal transcriptional programs, which could lead to a sustained tumor state [12]. Hence, TFs have been considered potential cancer therapeutic targets [13]. TF dysregulations could be due to cancer-specific signaling cascades that perturb TF expression or changes in the corresponding gene structure via chromosomal translocations/point mutations [14]. Some evidence of cancer-specific AS effects on TFs has been documented [10, 15]. For example, AS-induced alternative protein isoforms of T Cell Factor 4 (*TCF*4) have been implicated in proliferations of non-small cell lung cancer cells [16] and hepatocellular carcinoma [17]. Other examples are reviewed elsewhere [10]. Despite existing evidence, a comprehensive systematic analysis of how cancer-specific AS affects TFs has been missing. Such an analysis could reveal how TF-affecting AS events drive cancer progression, which could then be used to identify therapeutic targets and strategize drug design [18].

In this study, TCGASpliceSeq was used to systematically explore the effect of cancer-specific AS events across all known 1639 human TFs. 15 cancer types with AS data for enough paired (cancer and healthy) patient samples were used. For each cancer type, AS events showing statistically significant differences between paired cancer and normal samples were considered as perturbed. 2170 perturbed TF-related splicing events (PTSEs), i.e., events that affect TFs and show significant AS pattern differences between normal and paired cancer samples, covering 727 TFs across 14 cancer types, were found. Comparisons across cancer types revealed several unique cancer type-specific, as well as common, PTSEs. In 205 of the 727 (~26%) TFs, at least one PTSE affects TF functional domains. This strongly suggests that cancer-specific AS events could have a functional impact on TFs. Additionally, we found that the expression patterns of >100 PTSEs covering >70 of the 727 TFs have significant correlations with the existing sample-matched TF footprinting data [19], implying a possible dependence of the DNA binding and gene regulatory activities of the affected TFs on the corresponding AS events. Utilizing existing cancer dependency data [20], we also found that many (40) TFs affected by PTSEs are also essential for cancer progression in cancer cell lines. Lastly, using a recent study that experimentally characterized the functions of TF isoforms [15], we found five PTSEs across 13 cancer types that could result in TF isoforms with altered transcriptional activities compared to their corresponding reference TFs. Overall, our findings highlight a large-scale, likely functional, impact of cancer-specific AS on TFs. The collected evidence could guide future experiments to understand cancer molecular mechanisms or identify therapeutic targets.

## Data and methods

### Alternative splicing data

From the TCGA database [21] (accessed Feb. 2025), 15 cancer types were selected, where each cancer type had at least 10 patients with primary tumor (cancer) and patient-matched control (healthy) samples (Table 1). For these paired samples, the corresponding AS data were downloaded from TCGASpliceSeq [5], which utilized the SpliceSeq method [22] to quantify AS events. SpliceSeq creates a reference model (i.e., splice graph) of a gene by assembling all of the gene’s protein-coding transcripts from the Ensembl database. In a splice graph, whole or partial exons (or simply “transcript elements”) are represented as nodes, and any two nodes are connected by an edge if the corresponding transcript elements are adjacent in at least one known transcript of the gene. A splice graph contains a pre-determined set of transcript elements (along with their genomic coordinates) of a gene. Given RNAseq data, SpliceSeq aligns its reads to the pre-determined splice graphs in order to quantify the spliced-in transcript elements (or corresponding AS events) of genes. The TCGASpliceSeq data contains information about seven types of AS events: Alternative Acceptor (AA), Alternative Donor (AD), Alternative Promoter (AP), Alternative Terminator (AT), Exon Skip (ES), Mutually Exclusive Exons (ME), and Retained Intron (RI). TCGASpliceSeq provides Percent Spliced In (PSI) values for only those AS events that are covered by at least eight RNA-seq reads; for the rest of the AS events, a “null” value is provided. Given a cancer type, we only considered those AS events further that had PSI values for at least 10 paired samples.

**Table 1:** Patient, sample, and AS event counts across the 15 cancer types.

Cancer type	TCGA cancer code	# of patients with paired samples	# of cancer samples	# of AS events with values for at least 10 paired samples
Bladder urothelial carcinoma	BLCA	19	406	42,728
Breast invasive carcinoma	BRCA	113	1094	60,084
Colon adenocarcinoma	COAD	41	458	49,085
Esophageal carcinoma	ESCA	13	180	44,765
Head and neck squamous cell carcinoma	HNSC	42	501	51,429
Kidney chromophobe	KICH	25	66	50,046
Kidney renal clear cell carcinoma	KIRC	72	533	57,410
Kidney renal papillary cell carcinoma	KIRP	32	290	52,324
Liver hepatocellular carcinoma	LIHC	50	371	43,740
Lung adenocarcinoma	LUAD	57	514	52,263
Lung squamous cell carcinoma	LUSC	49	501	57,089
Prostate adenocarcinoma	PRAD	52	497	55,900
Stomach adenocarcinoma	STAD	34	415	54,709
Thyroid carcinoma	THCA	59	505	56,911
Uterine corpus endometrial carcinoma	UCEC	23	545	36,572

For each cancer type, significantly perturbed AS events were detected as follows. For an AS event, the patients with PSI values for both normal and cancer samples were detected. Considering these patients, a two-sided paired Wilcoxon Signed Rank test was performed to compare PSI values from normal samples with the PSI values from the paired cancer samples using the “*wilcox.test*” function in *R*. The obtained p-values of AS events were corrected using the Benjamini-Hochberg procedure to obtain the corresponding q-values. An AS event was considered significantly perturbed if and only if (1) its q-value was <0.05 and (2) the difference between the mean of normal PSI values and the mean of cancer PSI values was >5%. Note that, for any cancer type, more than 80% of the corresponding AS events had differences between the variances of the PSI values in the cancer samples and the variances of the PSI values in the paired normal samples of less than 0.01.

### Transcription factors

#### Curation and mapping of genomic coordinates

All 1639 curated human TFs were obtained from Supplementary Table S1 of the Lambert et al. [11] study. For these TFs, canonical protein sequences were collected from UniProt [23]. Henceforth, by the term “UniProt protein sequence” or “protein sequence”, we mean the “canonical UniProt protein sequence”. To map the genomic coordinates of AS events from the TCGASpliceSeq database onto protein sequences, UniProt IDs and Ensembl transcripts were matched using UniProt. Because one TF can have multiple Ensembl transcripts, one transcript per TF was selected as follows. Given a TF, its protein sequence was pairwise globally aligned with each of the corresponding Ensembl transcripts. Protein sequences of the transcripts were constructed using the release 113 of Genome Reference Consortium Human Build 37 (GRCh37) Ensembl GTF file “Homo_sapiens.GRCh37.dna.primary_assembly.fa.gz”. The transcript that matched the protein sequence with 100% identity and no gaps was selected. 1469 of the 1639 TFs with matched transcripts remained. Note that we used GRCh37 instead of GRCh38 because genomic coordinates of the AS events in the TCGASpliceSeq database are based on GRCh37. Nevertheless, we additionally mapped GRCh38 coordinates onto protein sequences using Ensembl transcript IDs selected above. Genomic coordinates of AS events provided by TCGASpliceSeq were then used to mark parts of the TF protein sequences affected by AS events.

**Fig. 1:**
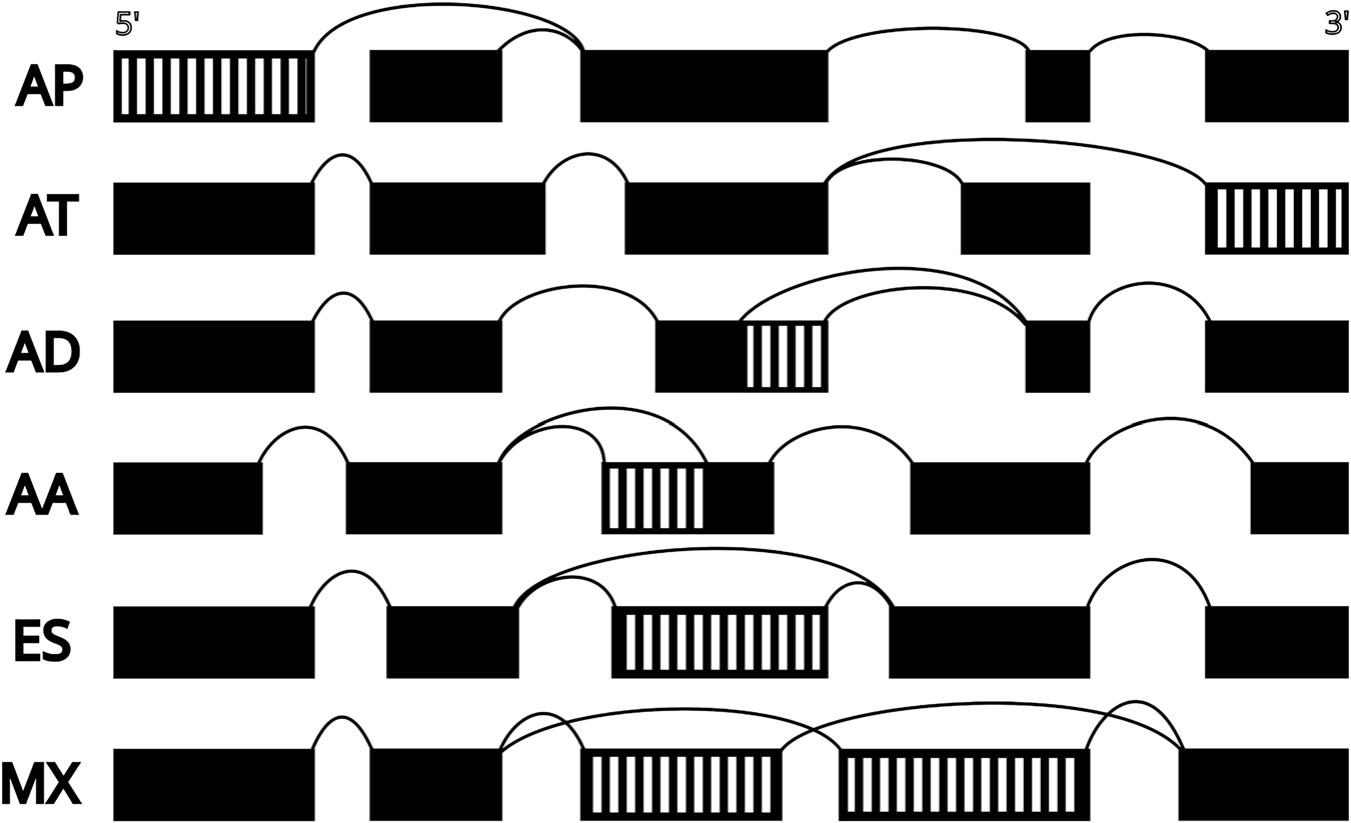
Schematic illustration of how AS affected exons (marked as striped boxes) were determined in this study. 1st row: for the AS event using the second exon as AP, the first exon was marked as affected. 2nd row: for the AS event using the second last exon as AT, the last exon was marked as affected. 3rd row: for the AS event using an alternative 3’ end of an exon (AD), the part of the exon beyond the alternative 3’ was marked as affected. 4th row: for the AS event using an alternative 5’ end of an exon (AA), the part of the exon before the alternative 5’ was marked as affected. 5th row: for the AS event skipping an exon (ES), the corresponding exon was marked as affected. 6th row: for the AS event involving two mutually exclusive exons (MX), both exons were marked as affected.

Specifically, for each of the six types of AS events, i.e., AA, AD, AP, AT, ES, and ME (Fig. 1), the following was done. Note that RI AS events cannot be mapped to protein sequences as they do not overlap with annotated protein-coding regions. For AA, AD, ES, or ME, all sequence positions of a TF within the start and stop genomic coordinates of the affected exon(s) of an AS event were marked as affected. For AP, all sequence positions of a TF before the start of the genomic position of the affected exon were marked as affected. For AT, all sequence positions of a TF beyond the stop genomic position of the affected exon were marked as affected.

#### Mapping of DNA-binding and effector domains

UniProt collects DNA-binding domain (DBD) information of proteins using PROSITE [25], Pfam [26], or SMART [27]. This information was used to annotate which protein sequence positions of TFs belong to a DBD region, as follows. Given a TF, if present, the following feature table entries from UniProt were considered as a DBD: (1) entries “DNA_BIND” or “ZN_FING” or (2) entry “DOMAIN” with domain names “bHLH”, “bZIP”, or “MADS-box”. Of the 1469 TFs with a matching Ensembl transcript (“Curation and mapping of genomic coordinates”), 1347 had at least one sequence position belonging to a DBD. TF effector domain (ED) information was taken from Supplementary Tables S2 and S3 of the Soto et al. [28] study. Soto et al. looked for ED evidence across 1639 human TFs in the literature and manually curated the largest collection of 924 EDs across 598 TFs. For these TFs, information about the UniProt protein sequence positions of EDs is provided. This ED information was used to annotate which protein sequence positions of TFs belong to an ED region. Of the 1469 TFs “Curation and mapping of genomic coordinates”, 538 had at least one sequence position belonging to an ED region, covering 846 EDs.

### tSNE application to represent cancer samples

Across all 15 cancer types, 2280 TF-related AS events were identified that had PSI values for at least 10 paired normal and cancer samples in each cancer type. Among these, 1025 were detected as a PTSE in at least one cancer type. PSI patterns of these 1025 AS events for all 6876 cancer samples across the 15 cancer types were used as input to the “*Rtsne*” package of R to obtain representations of the cancer samples.

### Assessment of over- or under-representation of DBD types in PTSEs

Among the 1347 TFs with DBD annotation (“Mapping of DNA-binding and effector domains”), 1464 occurrences of DBDs were found, which was taken as the background size. Among all PTSEs across all cancer types, 148 unique DBDs were present, which was taken as the sample size. For each of the 29 DBD-types perturbed in at least one PTSE, their counts in the sample and the background were used to compute the corresponding over-representations via the hypergeometric test to obtain 29 p-values. Similarly, for each of the 29 DBD-types, their counts in the sample and the background were used to compute the corresponding under-representations via the hypergeometric test to obtain 29 p-values. The 58 hypergeometric p-values were corrected using the Benjamini-Hochberg procedure to obtain the corresponding q-values.

### Alternative splicing events significantly associated with patient survival

Associations between AS events from TCGASpliceSeq and cancer patient survival were obtained from the OncoSplicing database (“SpliceSeq_info_Survival.csv.gz”) developed by Zhang et al. [29]. For a cancer type, OncoSplicing contains AS events that correlate with clinical outcomes according to the Cox proportional hazards regression. All AS events with survival association p-values of less than 0.05 (reported in the column named “Pvalue_HR” of “SpliceSeq_info_Survival.csv.gz”) were considered significant and selected for our study.

### Correlation of PSI values of PTSEs and TF footprinting

TF footprinting data were taken from Supplementary Table S6 of Corces et al. [19], which covers 404 TCGA cancer samples and 841 TFs across 23 cancer types. Corces et al. provide footprinting depth and flanking accessibility values for 1723 TF-DNA motif pairs, with multiple DNA motifs for a given TF. For a cancer type, a correlation between PSI values of a PTSE and footprinting values of a TF-DNA motif pair was measured if and only if there were at least 10 cancer samples for which the PTSE had a non-null PSI value in TCGASpliceSeq and its corresponding TF had footprinting data in Corces et al. At least 10 cancer samples were considered to retain power in the correlation analysis. 796 PTSEs across 12 cancer types fulfilled this criterion (Supplementary Table S1).

Given a cancer type, for each PTSE selected as per the above criterion, we measured the correlation between its PSI values and footprinting depth of the corresponding TF-DNA motif pairs. Overall, 12 cancer types resulted in 3766 correlation pairs. Similarly, we obtained 3766 correlation pairs corresponding to flanking accessibility. We calculated the empirical p-value based on data randomization, as follows. For each of the 3766*2 = 7 532 pairs, 100 random correlations were computed by randomly shuffling the PSI values and the footprinting data. The fraction of random correlations higher than the real correlation was taken as the empirical p-value. The resulting 7532 p-values were corrected using the Benjamini-Hochberg procedure to obtain the corresponding q-values. A *q*-value < 0.05 was considered significant.

### Dependency profiles of TFs associated with PTSEs

Dependency profiles of cancer cell lines were downloaded from the DepMap [20] portal (“https://depmap.org/portal/data_page/?tab=customDownloads”), which included all available cell lines and genes. These data cover 12 cancer types considered in our study: BLCA, BRCA, COAD, HNSC, KIRC, LIHC, LUAD, LUSC, PRAD, STAD, THCA, and UCEC. For these 12 cancer types, cell lines corresponding the following terminologies (in the order of the cancer types mentioned above) from the “lineage_3” column of the data was used: “Bladder Urothelial”, “Breast Invasive Carcinoma”, “Colorectal Adenocarcinoma”, “Head and Neck Squamous Cell Carcinoma”, “Renal Clear Cell Carcinoma”, “Hepatocellular Carcinoma”, “Lung Adenocarcinoma”, “Lung Squamous Cell Carcinoma”, “Prostate Adenocarcinoma”, “Stomach Adenocarcinoma”, “Thyroid Cancer”, “Endometrial Carcinoma”. 680 TFs covering at least one PTSE across the 12 cancer types had dependency data with respect to at least one cancer cell line, totaling 2 589 TF-cancer type combinations.

### Collection of the transcriptional activity data of TF isoforms

Of all 246 TFs covering 693 isoforms taken from Supplementary Data S1 Lambourne et al. [15], 223 TFs (covering 632 isoforms) matched with our data (“Transcription factors”). Amino acid sequences of these 632 isoforms were globally aligned with the protein sequences of TFs in our data. An alternate isoform was associated with a PTSE if and only if the parts of the TF protein sequence not affected by the PTSE had a one-to-one match with the isoform’s protein sequence. Eight PTSE-isoform pairs were detected across 13 cancer types. Five of the eight isoforms had transcriptional activity data in the Supplementary Data S3 of the Lambourne et al. study. These transcriptional activities were measured using a modified mammalian one-hybrid assay in HEK293T cells [15] with 3 replicates per isoform. For each isoform, the mean activity score was taken as the representative activity.

## Results

### Perturbed TF splicing events in individual cancer types

Given a cancer type, all AS events showing statistically significant PSI differences between paired normal and cancer samples were considered as perturbed AS events (“Alternative splicing data”). From these, all AS events corresponding to TFs (“Transcription factors”) were considered as PTSEs.

**Fig. 2:**
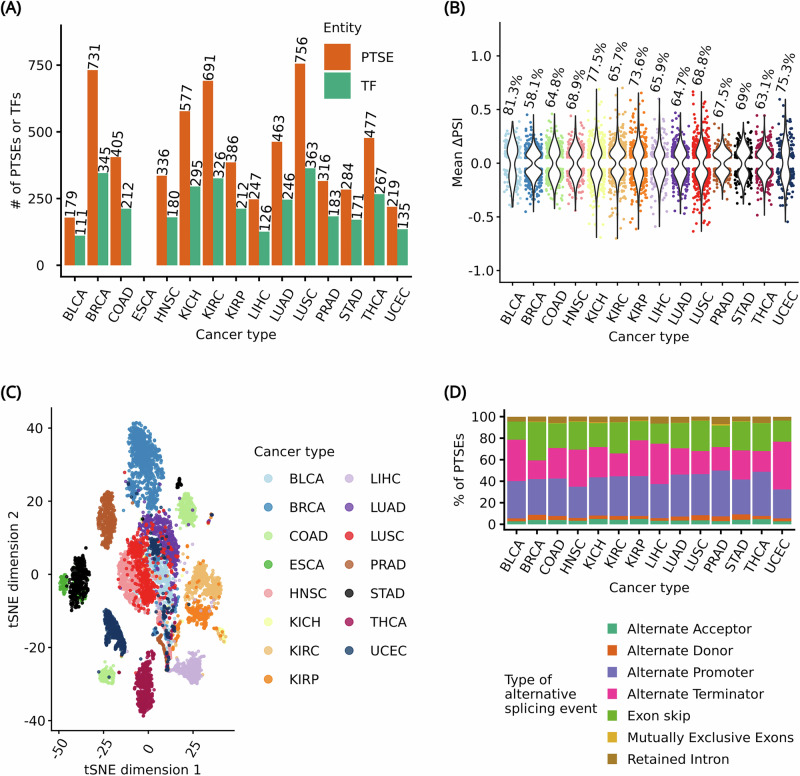
Cancer type-related PTSEs. **A** Number of cancer-type related PTSEs and the corresponding number of affected TFs. **B** Distribution of mean ΔPSI values of PTSEs. For a cancer type, the y-coordinate of a point (PTSE) represents the ΔPSI averaged over all patients with paired samples. The top of a violin bar represents the average (over all PTSEs of the corresponding cancer type) percentage of patients in which a PTSE is consistently higher/lower in cancer samples than in paired normal samples. **C** t-SNE visualization of the cancer samples of the entire TCGA cohort. **D** Percentage distribution of the types of AS events.

Over all cancer types, 727 TFs were affected by at least one PTSE. Many PTSEs were found for each cancer type, except for ESCA, which showed no perturbed AS event and thus no PTSE. No perturbed AS event for ESCA could be due to the low number of samples (13; see Table 1) that do not provide enough statistical power to detect significant differences between cancer and normal samples with the current q-value cutoff. The number of PTSEs showed large variations across cancer types, with the highest number (756) of PTSEs for LUSC (Fig. 2A). On average over all cancer types, ~1.8 PTSEs came from every TF, implying that no TF dominates PTSEs. PTSE numbers within cancer types do not seem to correlate with the corresponding numbers of paired normal and cancer samples (Table 1). For example, LIHC, LUAD, LUSC, and PRAD had similar numbers (50, 57, 49, and 52, respectively) of paired samples, but their PTSE numbers varied substantially: 247, 463, 756, and 316, respectively. Overall, these results indicate that not only are there significant TF AS differences between normal and cancer samples within a cancer type, but these differences also vary across cancer types.

For many PTSEs within a cancer type, their PSI values of cancer samples were higher/lower than their PSI values of the paired normal samples for a majority of patients (Fig. 2B). For example, the PSI values for the AS event *HMGA2_22880_AT* (here, “HMGA2” is the gene symbol, “22880” is the AS event number provided by the TCGASpliceSeq database, and “AT” is the type of AS event) were higher in cancer than normal samples for 52 out of the 57 paired samples of THCA with a mean ΔPSI of around 0.44 (Supplementary Table S2). Here, ΔPSI was defined as the difference between the PSI value of a cancer sample and the paired normal sample, with mean ΔPSI denoting the average ΔPSI over all paired samples of a given cancer type. The above results imply cancer type relevance of PTSEs. To further check whether this is the case, we used t-distributed Stochastic Neighbor Embedding (tSNE) to visualize cancer samples of the entire cohort of the considered cancer types, based on PSI patterns of all 1 025 AS events that were present in each of the 15 cancer types and were present as PTSEs in at least one cancer type (“tSNE application to represent cancer samples”). We found that cancer type-related samples were generally clustered together (Fig. 2C), implying cancer tissue specificity of the PSI patterns of PTSEs. Similar results have been shown using PSI patterns of all AS events in cancer [8, 9]. Interestingly, although we found large variations in PTSEs with respect to their numbers and PSI patterns across cancer types, the proportions of different types of AS events within PTSEs were similar across cancer types. That is, AP, AT, and ES dominate PTSEs (Fig. 2D), which has also been found in existing studies using all AS events [8, 9].

### Common and unique PTSEs of individual cancer types

**Fig. 3:**
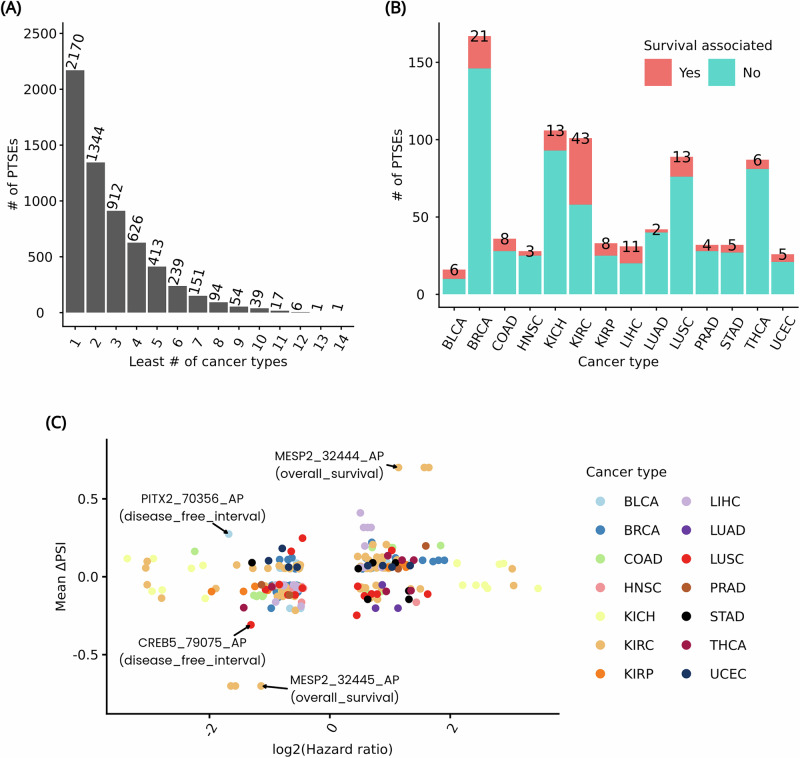
Common and unique PTSEs. **A** Number of PTSEs that occur in one or more cancer types. **B** Number of unique PTSEs of individual cancer types, with the number of such PTSEs that are uniquely associated with patient survival denoted on top of the bars. Information about the survival association was derived from Zhang et al. [29]. **C** Distribution of hazard ratios (x-axis) vs. ΔPSI values (y-axis) of the unique PTSEs of individual cancer types. For visualization purposes, one PTSE with >10 log2(hazard ratio) was left out.

Of the total 2170 PTSEs, a majority (~62%, i.e., 1344 PTSEs) occurred in more than one cancer type (Fig. 3A and Supplementary Table S3). 39 PTSEs occurred in at least 10 cancer types (Supplementary Fig. S1). An example PTSE is *PHD19_87401_AT* that affects the PHD Finger Protein 19 (*PHD19*) gene. This PTSE was upregulated in cancer samples in 12 cancer types, and corresponds to a known PHF19 isoform (hPCL3s) that is related to cancer progression of multiple cancer types [30–33]. We also found many unique PTSEs (826 in total) for individual cancer types that varied across cancer types, with BRCA having the most (167) and BLCA having the least (16) of them (Fig. 3B, C). Interestingly, at least one of the unique PTSE is survival-associated with the corresponding cancer type (“Alternative splicing events significantly associated with patient survival”) but not to any of the other cancer types (Supplementary Table S4). For example, PTSE *PITX2_70356_AP* affects the Pituitary homeobox 2 (*PITX2*) gene that results in the PITX2 isoform named PITX2D [34]. *PITX*2*_70356_AP* was upregulated in cancer samples compared to normal samples in BLCA with a mean ΔPSI of 0.27, and was found to be associated with the disease-free interval of BLCA with a hazard ratio of 0.31 (Supplementary Table S4). This means that an increase in the inclusion of this PTSE indicates a decreased risk of BLCA recurrence. PITX2 isoforms have been shown to be crucial for developmental processes, with PITX2D inhibiting the transcriptional activities of two other PITX2 isoforms by physically interacting with them [34]. This might explain why the increased expression of *PITX*2*_70356_AP*, which potentially leads to an increased expression of PITX2D, correlates with a decrease in the risk of BLCA recurrence. These results suggest that unique PTSEs could be relevant for understanding the molecular mechanisms of disease progression of individual cancer types.

### PTSEs perturb DNA-binding domains

DBDs are important functional components of TFs, which enable TFs to recognize and bind specific DNA sequences related to their target genes [11]. AS events affecting DBDs can impact the DNA-binding ability of the resulting TF isoforms. To capture this, we evaluated whether PTSEs affect DBD regions of TFs (“Transcription factors”).

**Fig. 4:**
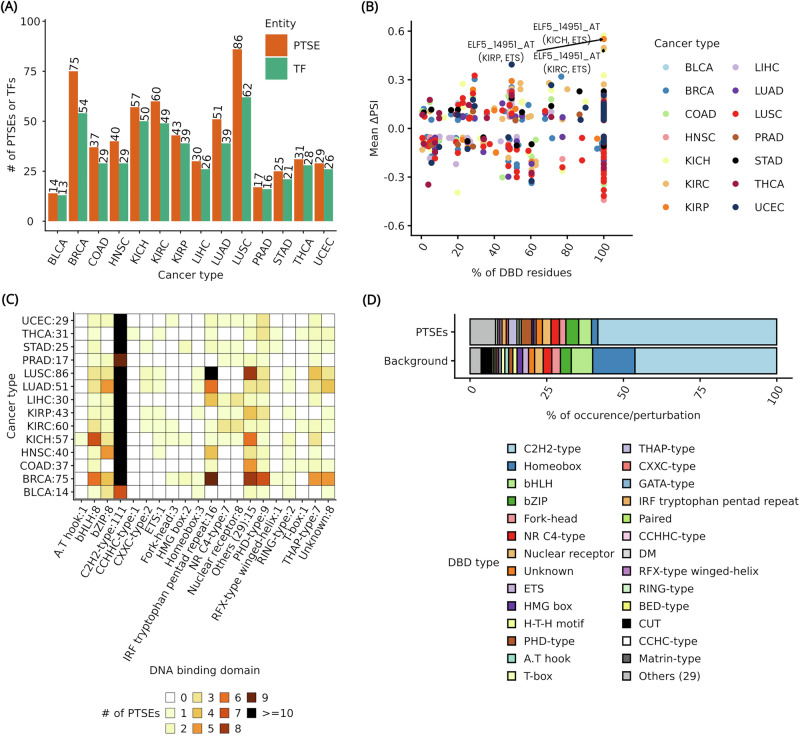
PTSEs that perturb DBDs. **A** Number of cancer type-related PTSEs that perturb DBDs. **B** Distribution of the percentage of DBD residues perturbed by PTSEs. Each point represents a PTSE (with the mean ΔPSI of the corresponding cancer type(s) on the y-axis) that results in a DBD perturbation (with the percentage of the perturbed DBD region on the x-axis). **C** Perturbed DBD types within PTSEs. The x-axis shows the names of DBD types and the corresponding number of PTSEs separated by a colon. For example, “bHLH:8” in the second column from left denotes that bHLH is perturbed in 8 PTSEs. The y-axis shows the cancer types and the corresponding number of PTSEs separated by a colon. For example, “BLCA:14” on the first row from the bottom, denotes that BLCA has 14 PTSEs. **D** Percentage distribution of the DBD types within all considered TFs (background) and those within PTSEs. For visualization purposes, 29 DBD types with counts of less than 5 in the background set of 1 347 TFs were grouped into one as “Others (29)”.

DBD regions of 136 TFs (i.e., ~10% of all 1347 TFs with DBD information were perturbed by at least one PTSE (Supplementary Table S5). Similar to the numbers of all PTSEs (Fig. 2A), the numbers of DBD perturbing PTSEs show large variations across cancer types, with the highest number (86) for LUSC and the lowest number (14) for BLCA (Fig. 4A). The proportions of different types of AS events within DBD perturbing PTSEs were dominated by AT and ES (supplementary Fig. S2). Of the 215 combinations of PTSE and the affected DBDs, 148 (~69%) affect large portions (>50%) of DBD regions (Fig. 4B). An example is the PTSE *ELF5_14951_AT* that removes 100% of the Erythroblast Transformation Specific (ETS) domain of E74-like transcription factor 5 (ELF5) and has higher PSI values in cancer samples than in normal samples of kidney cancer: KICH, KIRP, and KIRC (Supplementary Table S4). Interestingly, ELF5 has been shown to be a lineage-specific TF of principal cells that are key cells involved in kidney development [35].

Different DBD types show different perturbation patterns across cancer types (Fig. 4C). Some DBD types (i.e., bHLH, bZIP, C2H2-type, IRF tryptophan pentad repeat, Others(29), PHD-type, and THAP-type) were perturbed in multiple (>10) cancer types, while others (i.e., A.T hook and CCHHC-type) were perturbed in selected (<3) cancer types. We next evaluated whether certain DBD types are significantly over/under-represented within PTSEs. Specifically, we quantified the number of DBDs within PTSEs of any cancer type compared to the number of DBDs over all considered TFs, using the hypergeometric test (“Assessment of over- or under-representation of DBD types in PTSEs”). The proportions of DBDs among all considered TFs and those present in PTSEs were similar, with one significant exception (Fig. 4D). That is, the Homeobox domain was significantly under-represented (*q*-value < 10^–05^) in PTSEs. Interestingly, under-representation of the Homeobox domain in cancer was also observed in a recent study that detected 1350 TF-DNA interactions among 265 TFs and cloned promoters of 108 cancer-related genes [36]. These results could mean that the Homeobox domain is not only less relevant for cancer-related gene regulations in general, but it is also less affected by cancer-related AS events. Overall, the above results could reflect the underlying AS-induced shared and unique TF-DNA binding perturbations among cancer types, which could drive future experimental investigations to further explore the functional relevance of PTSEs in cancer phenotype development.

### PTSEs perturb effector domains

Besides DBDs, effector domains (EDs) also constitute important functional components of TFs that enable activation or repression of the target genes [28]. Thus, AS events affecting EDs could also impact TF functions. To capture this, we evaluated whether PTSEs affect ED regions of TFs “Transcription factors”.

**Fig. 5:**
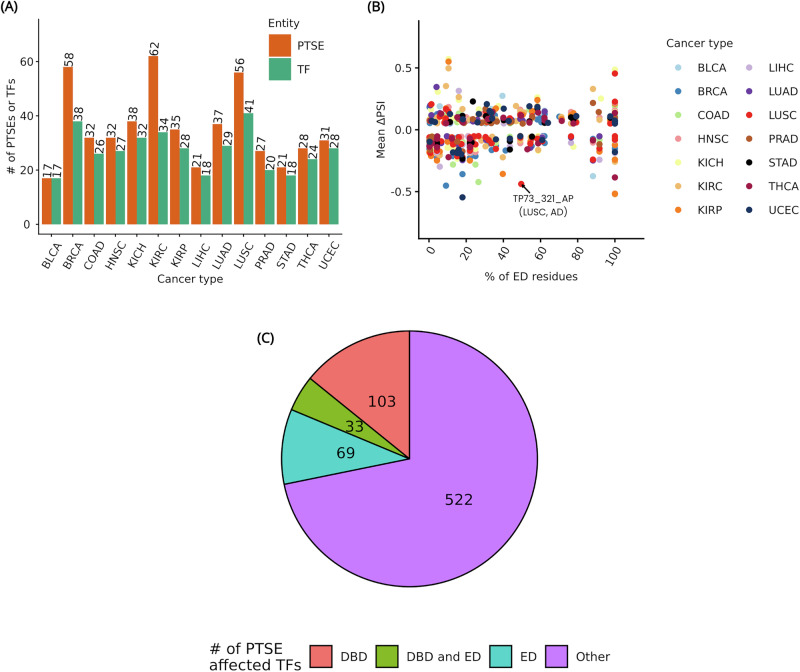
PTSEs that perturb EDs. **A** Number of cancer type-related PTSEs that perturb EDs. **B** Distribution of the percentage of ED residues perturbed by PTSEs. Each point represents a PTSE (with the mean ΔPSI of the corresponding cancer type(s) on the y-axis) that results in an ED perturbation (with the percentage of the perturbed ED on the x-axis). **C** Number of TFs with PTSE-induced perturbations in their DBD regions, ED regions, both DBD and ED regions, or other protein regions.

Similar to the numbers of DBD perturbing PTSEs (Fig. 4A), numbers of ED perturbing PTSEs show large variations across cancer types, with the highest number (62) for KIRC and the lowest number (17) for BLCA (Fig. 5A and Supplementary Table S6). The proportions of different types of AS events within ED perturbing PTSEs were dominated by AT, ES, and AP (supplementary Fig. S3). Of the 202 combinations of PTSE and the affected EDs, 62 ( ~ 31%) affect large portions (>50%) of ED regions (Fig. 5B), much less than the corresponding results for DBDs. An example ED perturbing PTSE is *TP73_321_AP* that removes ~50% of the activator domain (AD) of TP73 in LUSC and has higher PSI values in cancer samples than in normal samples of LUSC (Supplementary Table S6). *TP73_321_AP* leads to the ΔNp73 isoform of TP73, which is known to be overexpressed in tumors compared to normal conditions [37] and has been shown to fail in inducing apoptosis and cell cycle arrest [24].

Over all cancer types, 179 PTSEs perturb ED regions across 102 TFs (i.e., ~19% of all 538 TFs with ED information). This proportion is almost twice the proportion of TFs (~10%) whose DBD regions were perturbed by PTSEs. This could imply that PTSEs more frequently affect the transcriptional activity of TFs than their DNA-binding ability. Of all 727 TFs that were perturbed by at least one PTSE, 205 ( ~ 28%) involve a DBD or an ED, of which 33 TFs showed perturbations in both ED and DBD regions. These results could imply AS-driven functional perturbations of TFs in cancer.

### Alternative splicing patterns of PTSEs correlate with TF footprinting

To determine potentially AS-driven TF binding/activity patterns, we correlated the PSI values of PTSEs with the TF footprinting of the corresponding TFs in cancer samples of individual cancer types (“Correlation of PSI values of PTSEs and TF footprinting”). To do this, the chromatin accessibility data from Corces et al. [19] was used. Corces et al. used an assay for transposase-accessible chromatin using sequencing (ATAC-seq) to determine chromatin occupancy of TFs in TCGA cancer samples, quantified via “footprinting depth” and “flanking accessibility”. Intuitively, footprinting depth measures the inverse of the extent to which the binding of a TF to a chromatin region protects the corresponding TF-binding DNA motif from DNase [38]. Thus, low footprinting depth means high TF binding, and vice versa. In contrast, flanking accessibility measures the accessibility of the DNA region adjacent to TF-binding DNA motifs, which has been associated with TF activity [39]. Thus, high flanking accessibility could mean high TF activity, and vice versa.

**Fig. 6:**
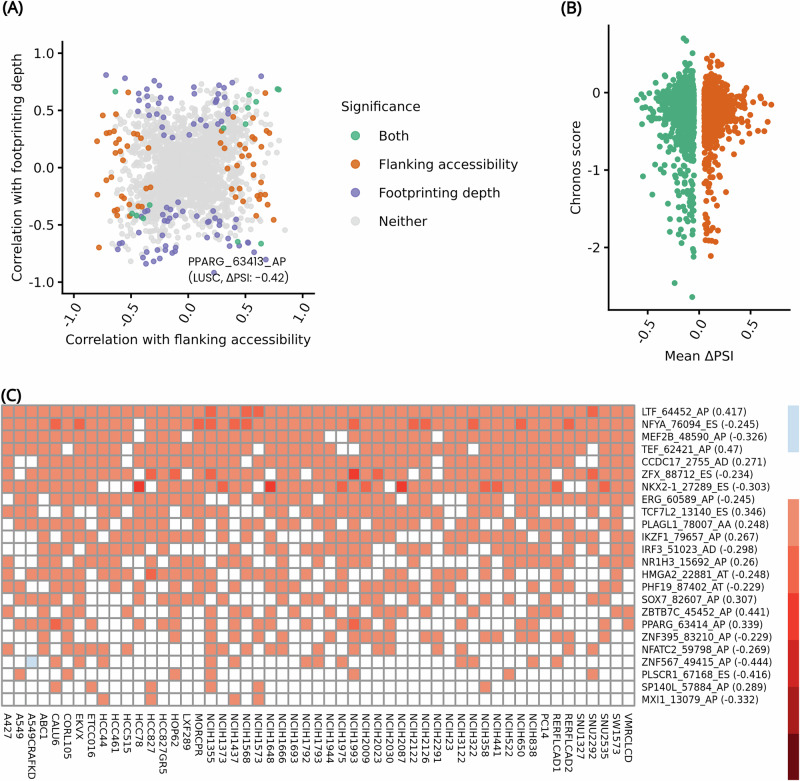
Footprinting and dependency characterization of PTSEs. **A** PTSEs correlated with footprinting. Each point represents one of the 1997 PTSE-cancer type combinations (Supplementary Table S1). Note that one PTSE-cancer type combination can have multiple footprinting depth/flanking accessibility values because of the presence of multiple DNA motifs for the corresponding TF (“Correlation of PSI values of PTSEs and TF footprinting”). In this case, the maximum correlation value (for each of the footprinting depth and flanking accessibility) was taken to generate this figure. **B** Distribution of dependency Chronos scores of all 2 589 TF-cancer type combinations (“Dependency profiles of TFs associated with PTSEs”). Each point represents a TF-cancer type combination, with the x-axis representing the maximum ΔPSI value among all PTSEs affecting the TF, and the y-axis representing the minimum Chronos score of the TF among all cell lines of the cancer type. **C** Chronos scores of TFs corresponding to PTSEs in LUAD, with the x-axis showing the cell line and the y-axis showing the top 1% of PTSEs with the maximum absolute mean ΔPSI value (denoted within brackets). For results corresponding to all cancer types, see Supplementary Fig. S4.

We found >100 PTSEs covering >70 TFs across >10 cancer types that showed significant (*q*-value < 0.05) correlation with footprinting depth, flanking accessibility, or both (Fig. 6A and Supplementary Table S7). Many of these PTSEs showed high mean ΔPSI values. For example, the PTSE *PPARG_63413_AP* affecting Peroxisome Proliferator-Activated Receptor Gamma (PPARG) in LUSC is significantly negatively correlated with footprinting depth and marginally significantly (FDR < 0.2) positively correlated with flanking accessibility. This could imply that, in LUSC, with an increase in the proportion of *PPARG_63413_AP* there is an increase in the DNA binding as well as the gene regulatory activity of PPARG. Because *PPARG_63413_AP* shows a mean ΔPSI of -0.42 in LUSC, DNA binding and gene regulatory activities of PPARG are perhaps higher in the normal tissue than in LUSC. Interestingly, previous in vivo [40] and in vitro [41] studies have shown that an increase in the PPARG activity slows down the tumor growth rate in non-small-cell lung cancer. Overall, this analysis identified PTSEs within cancer types whose inclusion rates are correlated with the footprinting data, potentially reflecting the underlying activities of the corresponding TFs.

### PTSEs include TFs showing cancer dependencies

Loss-of-function screens in cancer cell lines, e.g., using Clustered Regularly Interspaced Short Palindromic Repeats (CRISPR) [42], have identified genes essential for cancer cell line survival. Because many TFs have been associated with the sustained cancer cell state [12], we evaluated the extent to which the TFs within PTSEs show cancer dependency, using the CRISPR-Cas9 screening data from The Cancer Dependency Map (DepMap, Broad (2025). DepMap Public 25Q2. Dataset. depmap.org) [20] (“Dependency profiles of TFs associated with PTSEs”). Given a gene and a cancer cell line, DepMap provides gene perturbation effects based on the Chronos score. A Chronos score of 0 or above indicates that the gene is not essential for cancer cell proliferation, while a Chronos score below 0 indicates the gene is essential for cancer cell line proliferation, with the more negative value indicating more essentiality.

We found 680 TFs covered by PTSEs across 12 cancer types that had cancer dependency data for at least one cancer cell line (Fig. 6B). 658 of the 680 TFs showed a negative Chronos score in cell lines associated with at least one cancer type (Supplementary Table S8). 40 of these TFs showed high dependency score (Chronos score < –1) in at least one cancer cell line (Supplementary Table S8). An interesting example is the PTSE *NKX2-1_27289_ES* affecting Homeobox protein Nkx-2.1 (NKX2-1) in LUAD with a mean ΔPSI of -0.3 (Fig. 6C). NKX2-1 has two known isoforms [43], where the PTSE *NKX*2*-*1*_27289_ES* leads to a shorter protein isoform. Because the mean ΔPSI value of NKX2-1_27289_ES is negative, the shorter isoform NKX2-1 potentially shows higher prevalence in cancer samples than in normal samples. NKX2-1 is a lineage TF for lung [44]. Functional differences between the two isoforms have also been shown [45]. Because the minimum Chronos score of NKX2-1 is –1.4 (Supplementary Table S7), indicating its essentiality in cancer proliferation, perhaps it’s the higher prevalence of the shorter isoform that determines the cancer dependency of NKX2-1. Overall, this analysis identified TFs that are not only essential for cancer proliferation, but also showed significant differences between normal and paired cancer samples with respect to AS events.

### PTSEs could impact transcriptional activities of TFs

**Fig. 7:**
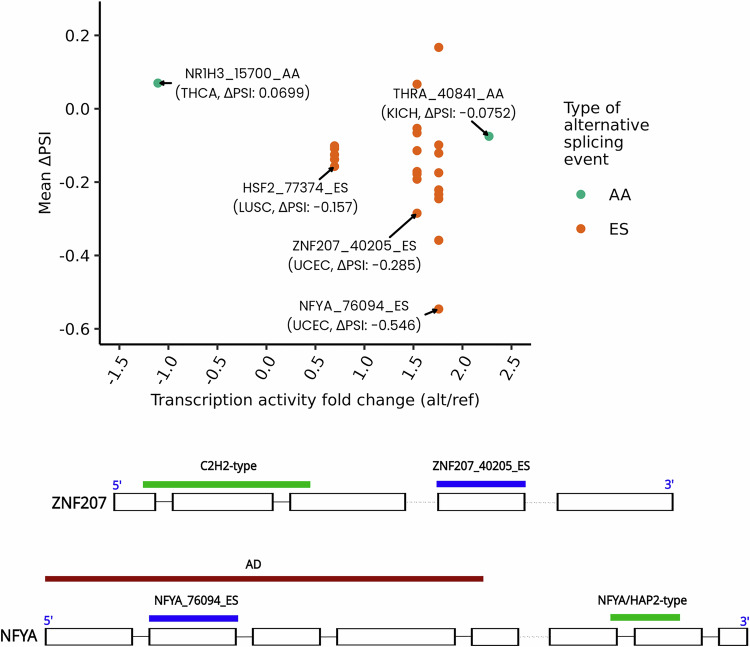
PTSEs leading to TF isoforms with altered transcriptional activities. In the upper panel, each point shows transcription activity fold change (alternative isoform vs. the reference TF) of a PTSE (x-axis) along with its mean ΔPSI value (y-axis) for the corresponding cancer type. The lower panel shows schematics of two selected TFs annotated with regions affected by PTSEs, as well as, if available, regions corresponding to DBDs and EDs.

To explore whether PTSEs could impact functions of the corresponding TFs, we evaluated whether the resulting isoforms show transcriptional activity changes as per the Lambourne et al. [15] study that experimentally evaluated altered functions of TF isoforms (“Collection of the transcriptional activity data of TF isoforms”). We found five PTSEs (three and two of AS types ES and AA, respectively) across 13 cancer types that matched five isoforms with the transcriptional activity data from Lambourne et al. [15]. All five isoforms showed altered transcriptional activity compared to the transcriptional activity of their corresponding reference TFs (Fig. 7). Of these, three (related to PTSEs ZNF207_40205_ES, NFYA_76094_ES, and THRA_40841_AA) showed an increase, one (related to PTSE HSF2_77374_ES) showed a decrease, and one (related to PTSE NR1H3_15700_AA) showed the opposite (repressing instead of activating) transcriptional activity compared to their respective reference TFs (Supplementary Table S9). Three of the five isoforms (related to PTSEs NFYA_76094_ES, HSF2_77374_ES, and NR1H3_15700_AA) perturb EDs (on average by 19%) of their corresponding TFs (Supplementary Table S5), which likely explains their altered transcriptional activity. These results provide stronger evidence for a potential functional impact of these PTSEs in the corresponding cancer types.

## Discussion

Many AS events showing significant differences between paired normal and cancer samples were found across 14 cancer types, affecting nearly 50% of known TFs with sufficient annotations. The number of such significant AS events varied considerably across cancer types. These variations could be related to variations in the expressions (and not necessarily mutation rates) of splicing factors in individual cancer types (Supplementary Figs. S5 and S6), although there are conflicting findings in the literature [46, 47]. Several of the affected TFs were found to be common in multiple cancer types, and many others were uniquely affected in a cancer type. These results highlight the pan-cancer significance of aberrant TF splicing in cancer. We found evidence of the impact of cancer-specific AS events on the DNA-binding and gene regulatory (activating/repressing) abilities of TFs. Further evidence for the functional impact of cancer-specific AS events on TFs was found by exploring whether the affected TFs were essential for cancer cell line proliferation.

Quantification of the differential usage of transcripts, rather than individual AS events, between paired normal and cancer samples could more accurately identify the involved protein isoforms. However, reconstruction of exact transcripts using short-read RNASeq data is non-trivial because a single gene could have multiple splice junctions that could lead to multiple different transcripts. Additionally, there is no single best algorithm to align partial transcripts to a reference genome [48]. Utilizing long-read RNASeq data could rectify these limitations of short-read RNASeq data. Different algorithms to detect and quantify AS events have been proposed in the literature, e.g., SpliceSeq [22] and SplAdder [49]. This work is based on the TCGASpliceSeq database, which uses the SpliceSeq algorithm to quantify AS events. Future work could evaluate whether these findings are robust across different algorithms.

Overall, our findings could be used to understand AS-driven transcriptional misregulations in cancer, which could guide the identification of novel therapeutic targets. In particular, the AS events leading to TF isoforms with altered transcriptional activities, as verified by Lambourne et al. [15] (“PTSEs could impact transcriptional activities of TFs”), could be further investigated. AS events affecting TFs with high cancer dependency scores (“PTSEs include TFs showing cancer dependencies”) could imply that the cancer cell lines showing proliferation dependency on these TFs are reliant on alternate isoforms. Such cases also merit further investigation. Gene regulatory associations based on gene-level expressions of TFs have provided interesting insights into cancer [50]. Our results show that there is a large-scale potential functional impact of cancer-specific AS events on TFs, which could perturb gene regulatory associations. Future inference of gene regulatory associations in cancer could benefit from considering expressions of TFs not just on the gene level but also on the isoform level.

## Supplementary information

Supplementary Materials

Supplement material.

## Data Availability

All data were taken from public databases, and the relevant links have been provided within the manuscript. The code to reproduce the work is publicly available at 10.5281/zenodo.19336347. The data extracted from the public databases for reproducing this work is available at 10.5281/zenodo.19335871.
