# Supplementary material for: Aberrant splicing in human cancer shows possible functional impact on transcription factors
Source: Oncogene. 2026 Jul 3;45(32):3287–98. doi: 10.1038/s41388-026-03876-9 (PMC13433238; doi:10.1038/s41388-026-03876-9)
Supplement: Supplementary file 1 [file 41388_2026_3876_MOESM2_ESM.pdf]

Supplement material for: Aberrant splicing in human cancer shows possible functional impact on transcription factors

Khalique Newaz[1,*], Olga Tsoy[1,2], and Jan Baumbach[1,3]

[1]Institute for Computational Systems Biomedicine, University of Hamburg, Germany
[2]Department of Computer Science, Bioinformatics, Vrije Universiteit Amsterdam, Amsterdam, The Netherlands
[3]Department of Mathematics and Computer Science, University of Southern Denmark, Odense, Denmark

* Corresponding author, khalique.newaz@uni-hamburg.de

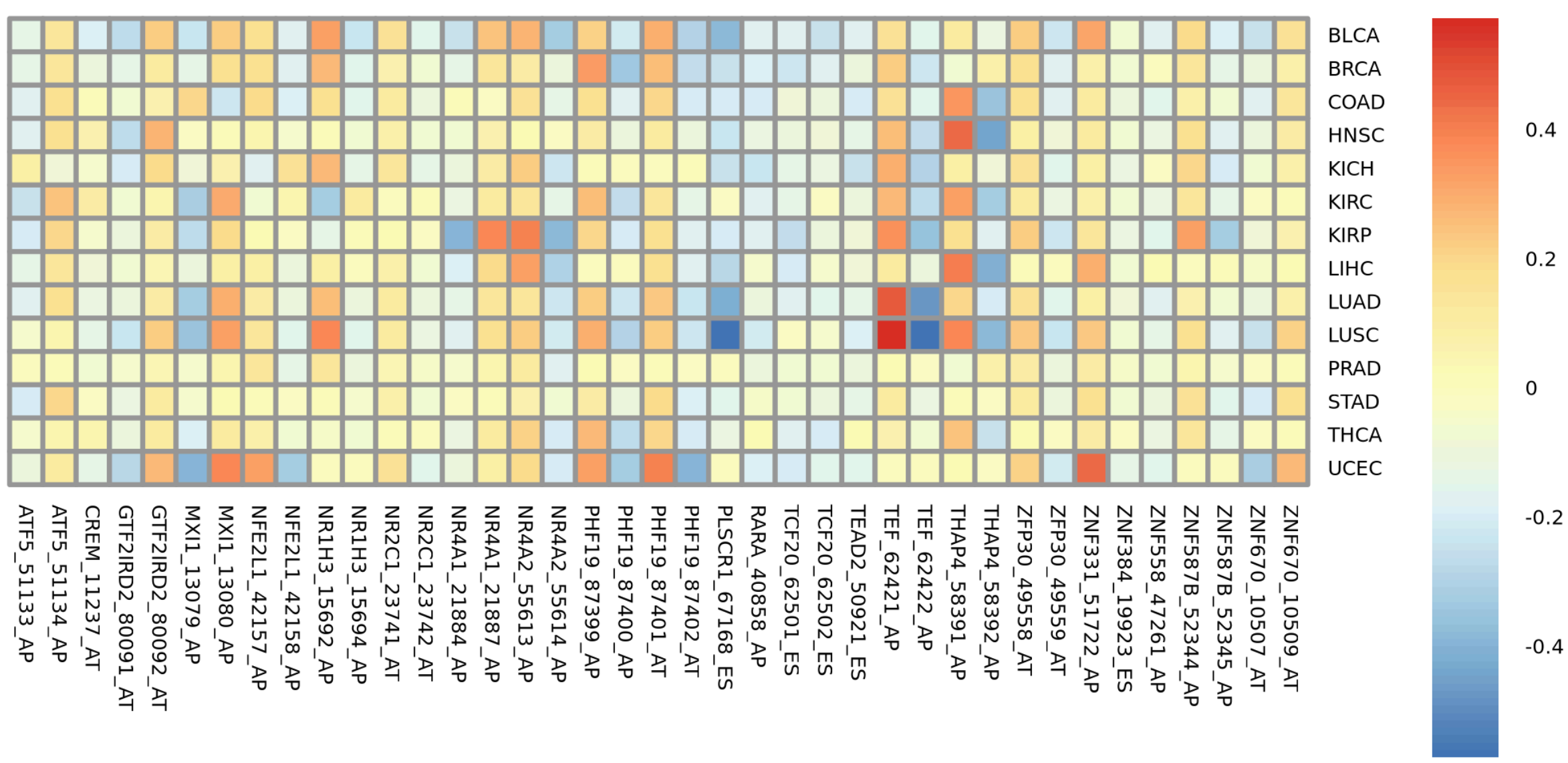

Supplementary Figure S1. Mean ΔPSI of the 39 PTSEs (y-axis) present in at least 10 cancer types (x-axis).

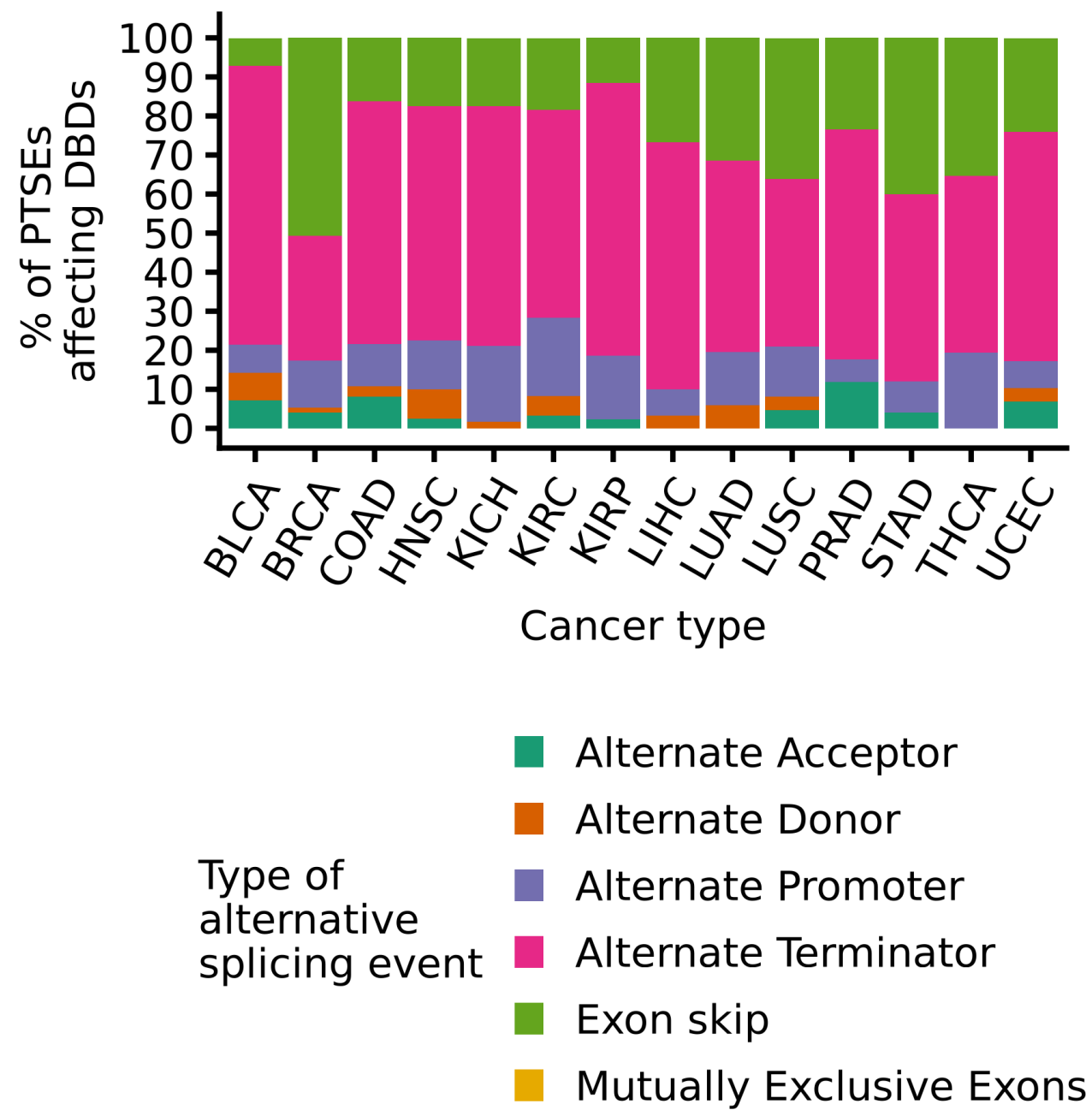

Supplementary Figure S2. Percentage distribution of the types of AS events within DBD perturbing PTSEs.

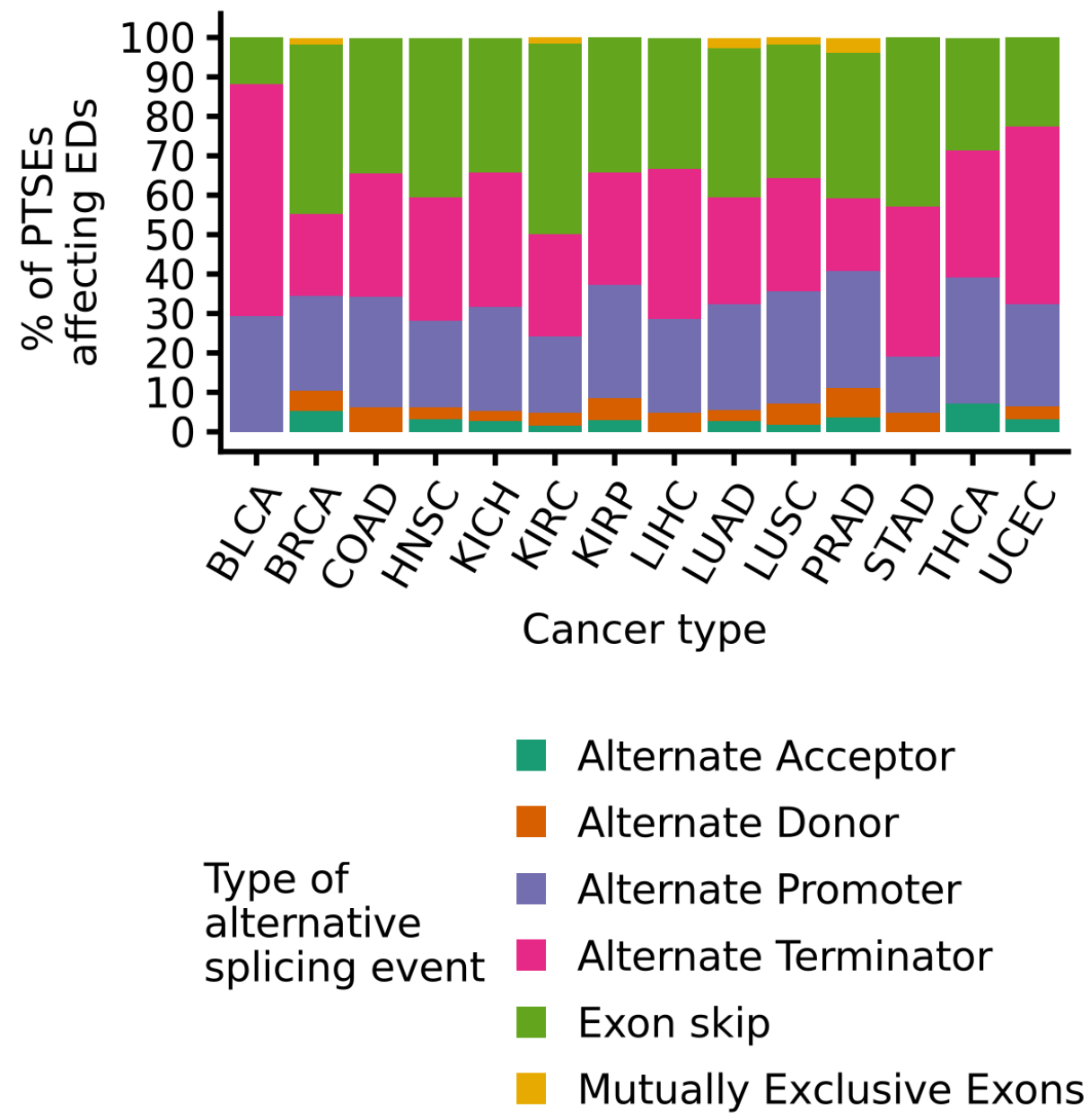

Supplementary Figure S3. Percentage distribution of the types of AS events within ED perturbing PTSEs.

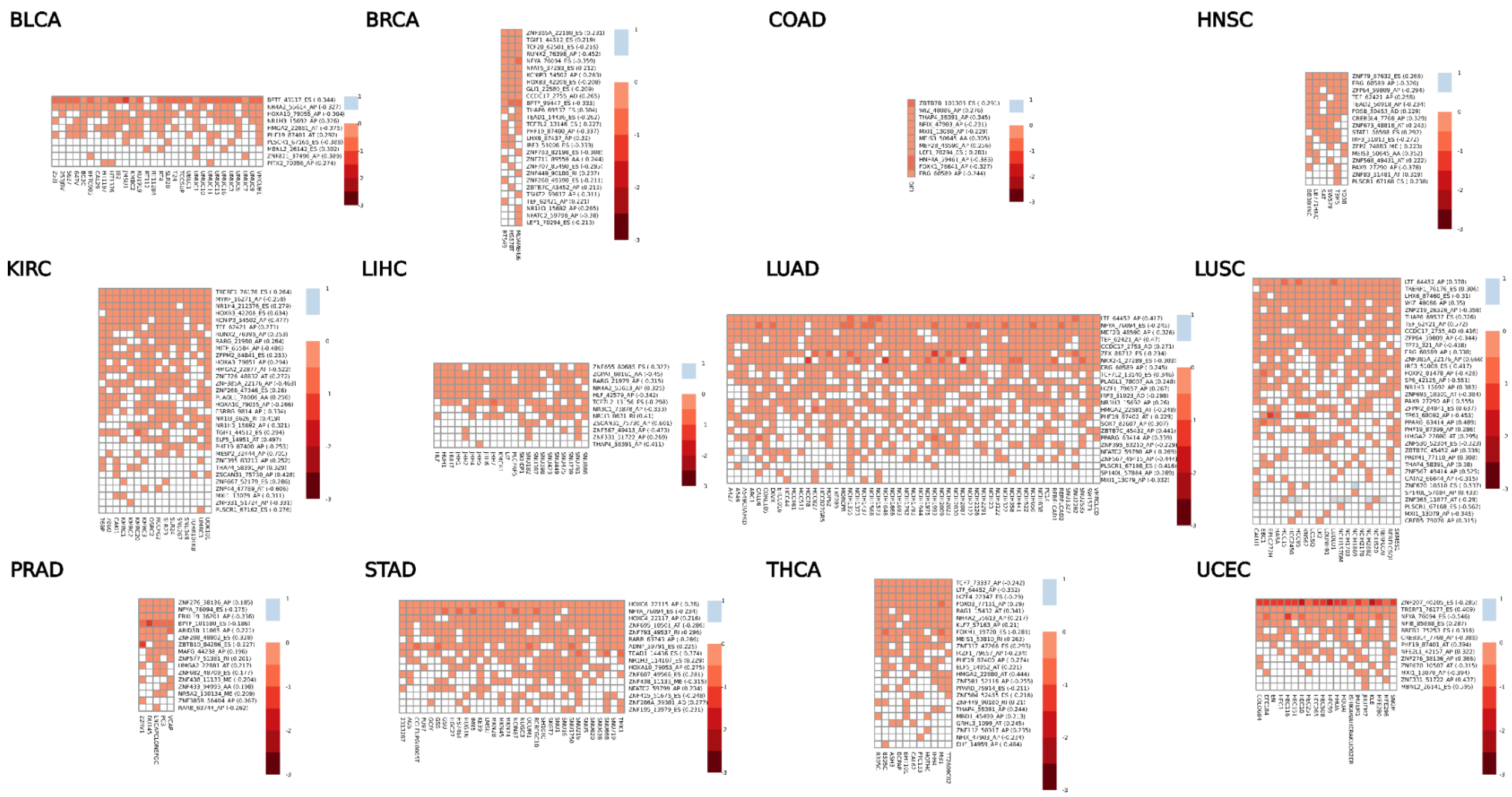

Supplementary Figure S4. Chronos scores of TFs corresponding to PTSEs in individual cancer types, with x-axis showing the cell line and the y-axis showing the top 1% of PTSEs with maximum absolute mean ΔPSI value (denoted within brackets).

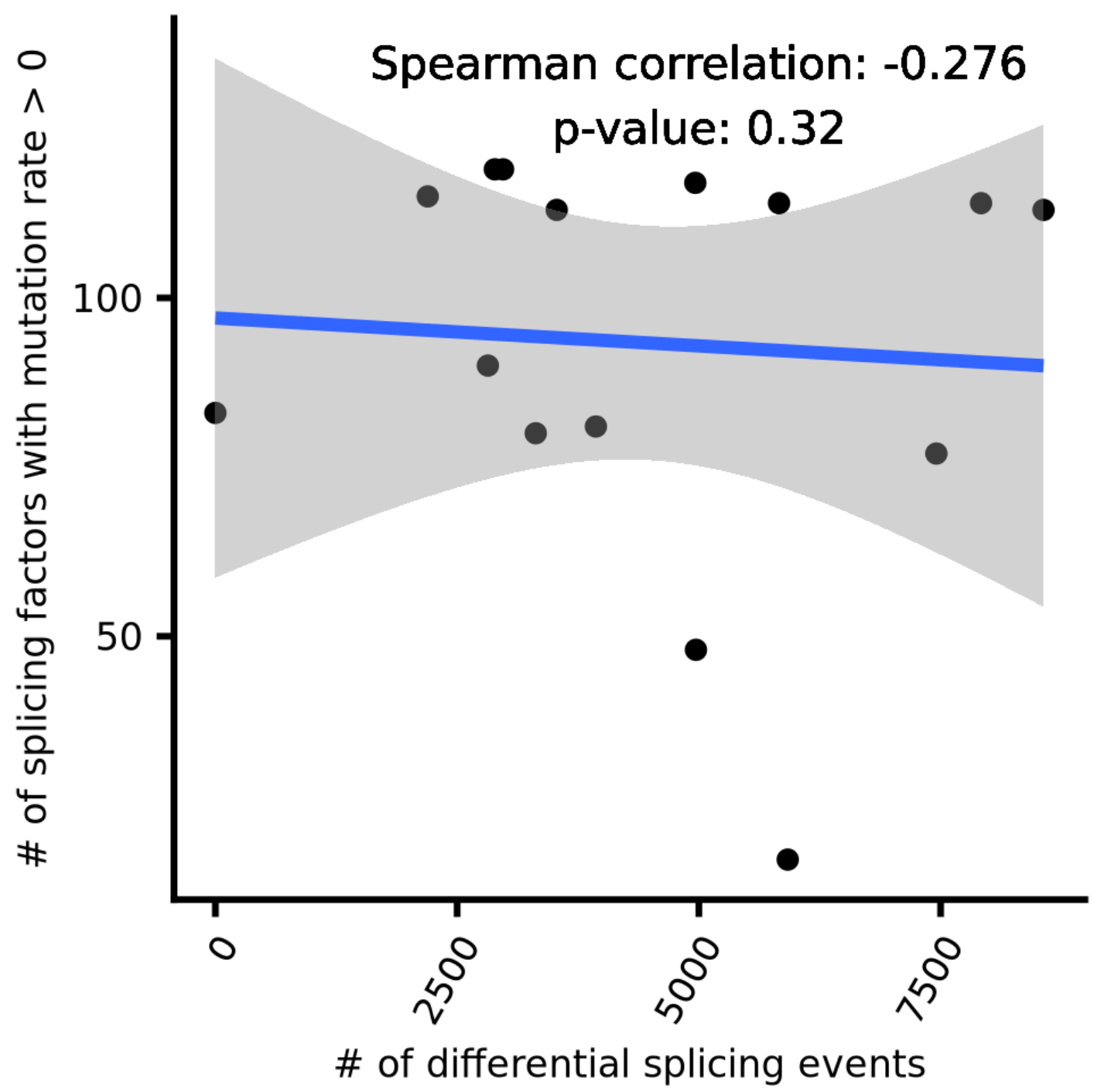

Supplementary Figure S5: Correlation between the numbers of splicing factors with non-zero mutation rates in different cancer types from Seiler et al.[1] and the numbers of significantly perturbed AS events of individual cancer types identified in our study. The mutation rate data were taken from the Supplementary Table S1 of the Seiler et al. study.

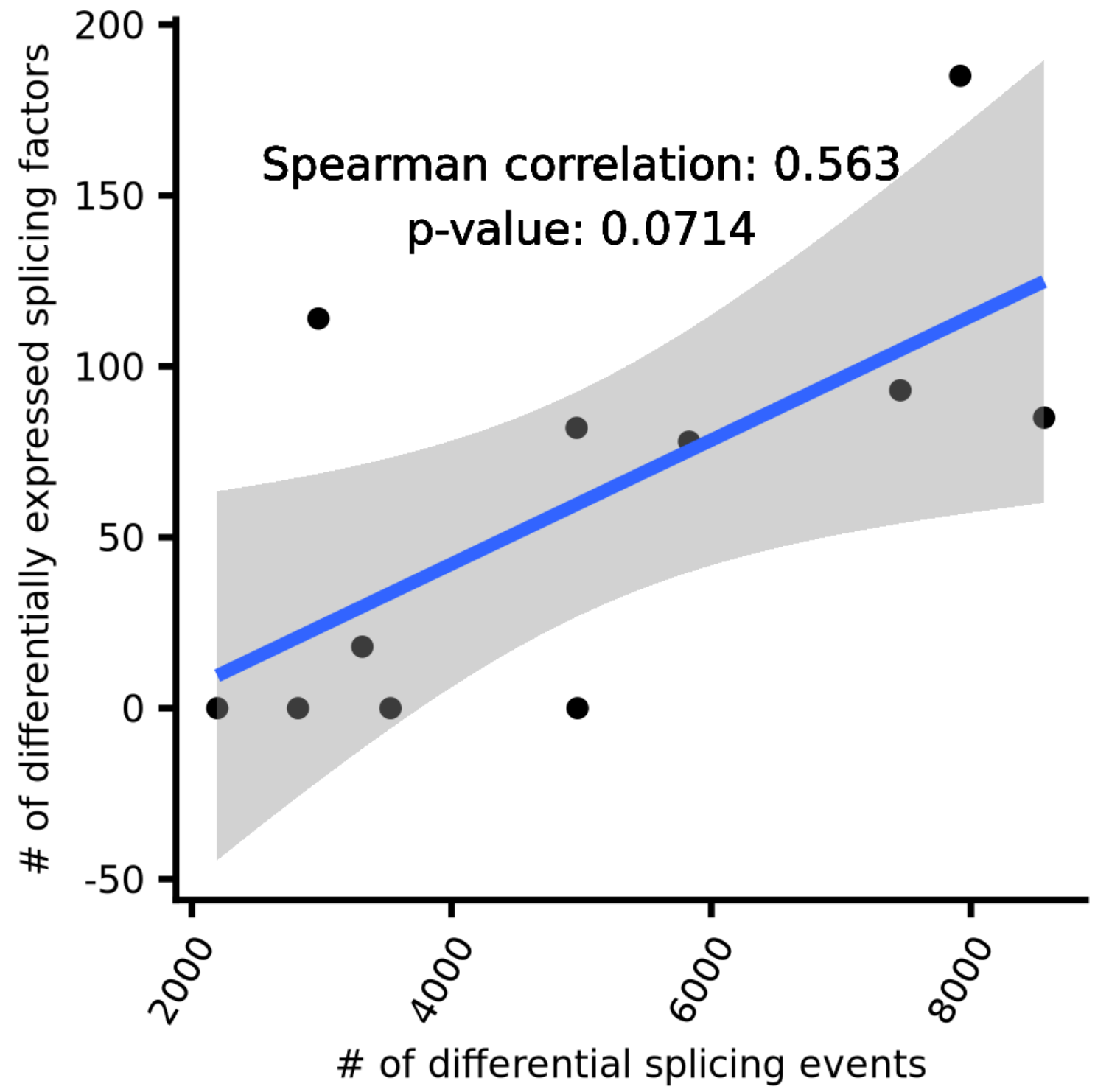

Supplementary Figure S6: Correlation between the numbers of differentially expressed (cancer vs. normal samples) splicing factor genes from Sveen et al.[2] and the numbers of significantly perturbed splicing events of individual cancer types identified in our study. The numbers of differentially expressed splicing factor genes were taken from the Supplementary Table S2 of the Sveen et al. study.

Supplementary Table S1: Overlap between TCGA SpliceSeq and Corces et al.[3].

TCGA cancer code	# of cancer samples	# of PTSEs with PSI values for at least 10 cancer samples	# of TFs within PTSEs with PSI values for at least 10 cancer samples
BLCA	10	66	41
BRCA	74	316	138
COAD	38	174	91
KIRC	16	244	117
KIRP	33	150	84
LIHC	17	93	37
LUAD	20	203	102
LUSC	16	293	134
PRAD	26	123	67
STAD	21	107	65
THCA	14	143	77
UCEC	13	85	52

Supplementary Table S2: List of cancer type-related PTSEs.

Supplementary Table S3: List of PTSEs and their cancer type occurrences.

Supplementary Table S4: List of PTSEs uniquely occurring in a cancer type.

Supplementary Table S5: List of DBD-perturbing PTSEs.

Supplementary Table S6: List of ED-perturbing PTSEs.

Supplementary Table S7: Correlation of PSI values of PTSEs with footprinting data.

Supplementary Table S8: Cancer dependency information of PTSE-related TFs.

Supplementary Table S9: List of PTSEs leading to TF isoforms with altered transcriptional activity as per Lambourne et al.[4].

References

[1] M. Seiler *et al.*, “Somatic mutational landscape of splicing factor genes and their functional consequences across 33 cancer types,” *Cell Rep.*, vol. 23, no. 1, pp. 282–296.e4, Apr. 2018.

[2] A. Sveen, S. Kilpinen, A. Ruusulehto, R. A. Lothe, and R. I. Skotheim, “Aberrant RNA splicing in cancer; expression changes and driver mutations of splicing factor genes,” *Oncogene*, vol. 35, no. 19, pp. 2413–2427, May 2016.

[3] M. R. Corces *et al.*, “The chromatin accessibility landscape of primary human cancers,” *Science*, vol. 362, no. 6413, p. eaav1898, Oct. 2018.

[4] L. Lambourne *et al.*, “Widespread variation in molecular interactions and regulatory properties among transcription factor isoforms,” *Mol. Cell*, vol. 85, no. 7, pp. 1445–1466.e13, Apr. 2025.
